# Plant-microbe rhizosphere interactions mediated by *Rehmannia glutinosa* root exudates under consecutive monoculture

**DOI:** 10.1038/srep15871

**Published:** 2015-10-30

**Authors:** Linkun Wu, Juanying Wang, Weimin Huang, Hongmiao Wu, Jun Chen, Yanqiu Yang, Zhongyi Zhang, Wenxiong Lin

**Affiliations:** 1 Key Laboratory of Biopesticide and Chemical Biology, Ministry of Education, Fujian Agriculture and Forestry University, Fuzhou 350002, Fujian, P. R. China; 2 College of Life Sciences, Fujian Agriculture and Forestry University, Fuzhou 350002, Fujian, P. R. China; 3 Fujian Provincial Key Laboratory of Agroecological Processing and Safety Monitoring, College of Life Sciences, Fujian Agriculture and Forestry University, Fuzhou 350002, Fujian, P. R. China

## Abstract

Under consecutive monoculture, the biomass and quality of *Rehmannia glutinosa* declines significantly. Consecutive monoculture of *R. glutinosa* in a four-year field trial led to significant growth inhibition. Most phenolic acids in root exudates had cumulative effects over time under sterile conditions, but these effects were not observed in the rhizosphere under monoculture conditions. It suggested soil microbes might be involved in the degradation and conversion of phenolic acids from the monocultured plants. T-RFLP and qPCR analysis demonstrated differences in both soil bacterial and fungal communities during monoculture. Prolonged monoculture significantly increased levels of *Fusarium oxysporum*, but decreased levels of *Pseudomonas* spp. Abundance of beneficial *Pseudomonas* spp. with antagonistic activity against *F. oxysporum* was lower in extended monoculture soils. Phenolic acid mixture at a ratio similar to that found in the rhizosphere could promote mycelial growth, sporulation, and toxin (3-Acetyldeoxynivalenol, 15-O-Acetyl-4-deoxynivalenol) production of pathogenic *F. oxysporum* while inhibiting growth of the beneficial *Pseudomonas* sp. W12. This study demonstrates that extended monoculture can alter the microbial community of the rhizosphere, leading to relatively fewer beneficial microorganisms and relatively more pathogenic and toxin-producing microorganisms, which is mediated by the root exudates.

*Rehmannia glutinosa* Libosch, a perennial herbaceous plant, is highly valued in traditional Chinese medicine. It provides treatments for endocrine, cardiovascular, nervous system, and immune system ailments^1^. However, extended monoculture of *R. glutinosa* results in a significant decline in the biomass and quality of the underground tubers which are of greatest medicinal value^2^. Fields used for *R. glutinosa* production are typically replanted every 15–20 years^3^. The causes of problems associated with extended monoculture of *R. glutinosa* have become a research priority in China.

Studies of consecutive monoculture problems (CMP) have focused on deficiency of soil nutrients and the autotoxicity of allelochemicals in root exudates^4^. Extended monoculture of *R. glutinosa* did not lead to decreased soil available nutrients, and fertilization did not eliminate replant disease or improve plant growth^5^. Bramley *et al*.^6^ found no consistent effect of time on soil chemical properties in sugarcane monocultures. Besides, filter paper bioassays enriched with a single chemical were inadequate for evaluating the allelopathic effects and ecological roles of root exudates in soil because these assays exclude effects of soil chemical properties and microbial communities^7^. More attention has focused on the root-associated microbial community, referred to as the “second genome” of the plant which is vital for plant health^8^. Soil ecosystem functioning is governed largely by microbial dynamics of the rhizosphere since microbial community composition and diversity affect geochemical cycles, humus formation and degradation, soil structure and biological interactions^9^.

Aboveground performance of plants is closely associated with the changes in belowground microbial communities^10^,^11^. Using 16S rRNA-based microarray analysis coupled with cloning sequences and quantitative PCR (qPCR), Sanguin *et al*.^12^ demonstrated changes in the rhizosphere bacterial community associated with take-all decline in wheat monoculture. Plants are able to modify their rhizosphere microbiome because of the selective effect of root exudates on different microbial species^13^,^14^,^15^. Qu *et al*.^16^ added two phenolic acids, phenol 2,4-di-tert-butylphenol and vanillic acid, detected in soybean root exudates, to soil and found these acids, especially vanillic acid, had a significant impact on microbial communities and soybean monoculture problems. Zhou *et al*.^17^ found no direct phytotoxic effects of root exudates in a monoculture regime but there were indirect affects on the growth of monocultured cucumber via changes in the soil microbial community. Previous studies demonstrated that monocultured *R. glutinosa* releases phenolic acids such as 4-hydroxybenzoic, vanillic, salicylic, and ferulic acid into the rhizosphere^18^. However, little is known about microbial community changes in the rhizosphere of *R. glutinosa* under monoculture and the ecological effects of the phenolic acids in root exudates. In this study, we hypothesized that the poor growth of *R. glutinosa* under extended monoculture might result from the altered microbial community in the rhizosphere mediated by root exudates.

Previous research on soil ecosystems was limited by the complex interactions among soil physical, chemical, and biological components^19^. The terminal restriction fragment length polymorphism (T-RFLP) technique provides a better understanding of the structure and diversity of microbial communities because of its high reproducibility and fidelity and automation potential^20^,^21^. In this study, the responses of both bacterial and fungal communities in rhizosphere soil from a four-year field trial were analyzed by T-RFLP combined with quantitative PCR technique. Several microbes closely associated with extended monoculture problems were identified and isolated for a plant-microbe interaction study. Data from this study can help explain the ecological links between above and below ground biota and the effect of root exudates on the selection of soil microbes. This study will also provide useful information on indigenous microbial populations with potential application to soil remediation and improvement.

## Results

### Above and belowground morphology of *R. glutinosa*

**Figure 1:**
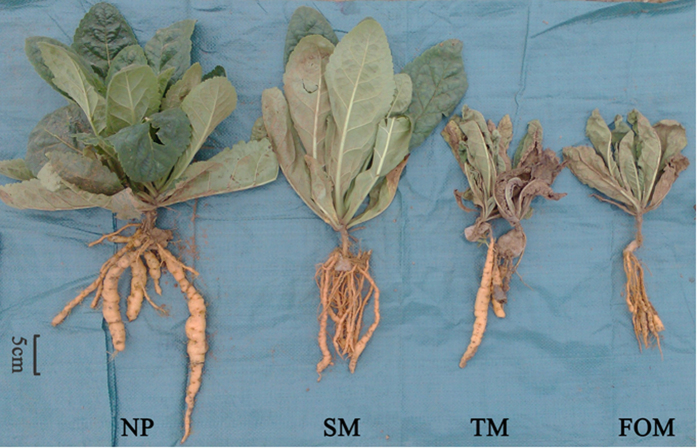
Photographs of above and below ground components of *R. glutinosa* under one-year, two-year, three-year and four-year consecutive monoculture.

Both above and belowground biomass of *R. glutinosa* markedly declined from one- to four-year monoculture fields (Fig. 1). Compared to the newly planted, the monocultured plants displayed poorer growth with the belowground component producing narrow tubers containing large numbers of adventitious fibrous roots. Consecutive monoculture always results in earlier wilt of the above ground plant and lower or no yield of the below ground root tubers. These problems worsen with the increasing years of monoculture.

### Quantification of phenolic acids in culture medium and rhizosphere soil

Ten phenolic acid compounds including coumaric acid, protocatechuic acid, phthalic acid, *p*- hydroxybenzoic acid, vanillic acid, syringic acid, vanillin, ferulic acid, benzoic acid and salicylic acid were identified from the *R. glutinosa* root exudates grown in culture medium under sterile conditions. Quantitative analysis with HPLC showed that most phenolic acids increased with growth time of the tissue culture seedlings (Supplementary Fig. S1a). However, only eight of the phenolic acids, excluding coumaric acid and salicylic acid, were found in the rhizospheric soil of *R. glutinosa*. The levels of most rhizosphere phenolic acids did not increase with increasing years of monoculture (Supplementary Fig. S1b), suggesting that accumulation of root exudates and their possible autotoxicity might not be the direct cause for consecutive monoculture problems. We hypothesized that soil microbes might be involved in the degradation, utilization, and conversion of root exudates.

### Non-metric Multi-Dimensional Scaling (NMDS) and cluster analyses

We obtained 45 bacterial T-RFLP profiles out of soil DNA extracted from five different treatments with *Msp*I, *Hae*III, and *Alu*I enzymes (Supplementary Fig. S2) and 45 fungal T-RFLP profiles with *Alu*I, *Hinf*I and *Taq*I enzymes (Supplementary Fig. S3). Most T-RFs ranged between 35 and 650 bp.

**Figure 2:**
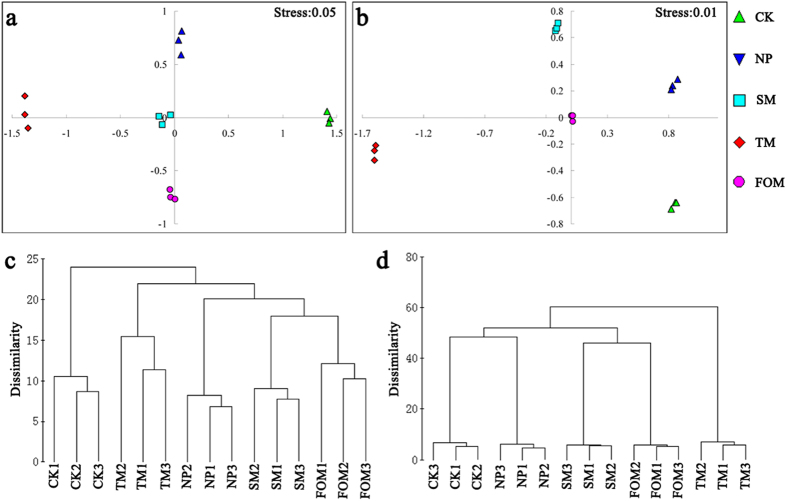
NMDS ordinations and cluster analysis of bacterial (**a,c**) and fungal (**b,d**) communities based on Bray-Curtis dissimilarities of the T-RFLP data consisting of the relative abundance of the terminal restriction fragments. CK, NP, SM, TM and FOM represent the control, newly planted, two-year, three-year and four-year consecutively monoculture soils, respectively.

For combined restriction enzymes, NMDS ordinations (relative abundance, logarithmic transformation, Bray-Curtis dissimilarity matrix) showed clear differences in both bacterial and fungal communities between the five different treatments (Fig. 2a,b). Kruskal’s stress values were less than 0.1, suggesting a good representation of the relationship between points in the matrix. Cluster analysis (relative abundance, logarithmic transformation, Bray-Curtis dissimilarity matrix) of bacterial and fungal communities showed that the consecutively monocultured soils (SM, TM, and FOM) were more similar and considerably different from the newly planted soils (NP), indicating a shift in the rhizosphere microbial community following extended *R. glutinosa* monoculture (Fig. 2c,d).

### Analysis of similarity (ANOSIM) and similarity percentage analysis (SIMPER)

Analysis of similarity (ANOSIM) of the T-RFLP data consisting of the relative abundance of T-RFs showed that both bacterial (ANOSIM Global R = 1.0, *P* = 0.001) and fungal (ANOSIM Global R = 1.0, *P* = 0.001) communities differed significantly among five different treatments. ANOSIM analysis based on the presence/absence of T-RFs also showed a significant difference among five treatments in both bacterial (ANOSIM Global R = 0.858, *P* = 0.001) and fungal (ANOSIM Global R = 1.0, *P* = 0.001) communities. When SM, TM, and FOM were combined as one sample and termed “consecutively monocultured soil (CM)”, SIMPER analysis (relative abundance of peaks) showed pairwise dissimilarities between NP and CM were 23.9% for the bacterial community and 64.37% for the fungal community, respectively. The top T-RFs with 20% cumulative contribution to the dissimilarity between NP and CM were listed as Table 1.

For the bacterial community, consecutive monoculture produced a significant increase in the relative abundance of fragments including *Hae*III 37 and 295 bp, *Alu*I 252 bp and *Msp*I 150 bp in the rhizosphere with the increasing years of monoculture, but a decrease in *Msp*I 293 bp, *Hae*III 205 bp, *Alu*I 249 and 72 bp. Assignation from PAT has identified the fragment *Alu*I 72 bp to be from *Pseudomonas* spp. in the class *GammaProteobacteria*. With regard to fungal community, T-RFs including *Alu*I 428 and 588 bp, *Hinf*III 65 bp were significantly higher in newly planted soil than in consecutively monocultured soil while T-RFs including *Taq*I 251 and 357 bp, *Hinf*III 304 bp were significantly higher in consecutively monocultured soil (*P* < 0.05). The experimental T-RFs were determined by the *in silico* T-RFs deduced from the ITS sequences from GenBank and 50 fungal isolates. Several T-RFs (i.e. *Hinf*III 304 bp and *Taq*I 251 bp) constituting the highest proportion of the fungal population in two or three-year monoculture soils were assigned as *Fusarium oxysporum* (Table 1).

### Abundance of *Pseudomonas* and *F. oxysporum* by quantitative PCR

**Figure 3:**
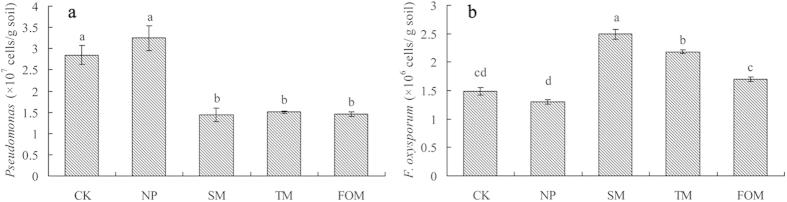
Quantification of *Pseudomonas* (a) and *Fusarium oxysporum* (b) in control (CK), newly planted (NP), two-year (SM), three-year (TM), and four-year (FOM) monoculture soils by quantitative PCR. Data are means ± standard errors (one-way analysis of variance, n = 4).

**Figure 4:**
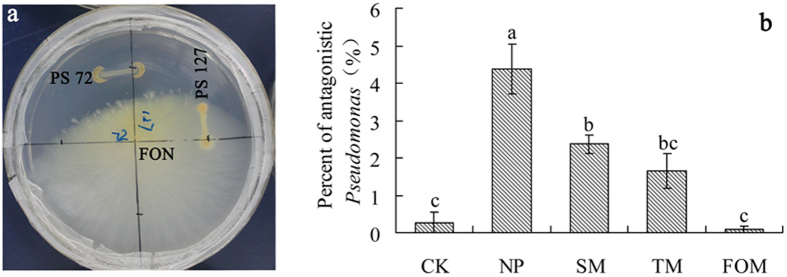
Relative abundance of *Pseudomonas* spp. with antagonistic activity against *F. oxysporum* in five different samples. (**a**) Strain 72 but not 127 has antagonistic activity against *F. oxysporum* (FON) on 9 cm diam Petri dishes. (**b**) CK, NP, SM, TM and FOM represent the control, newly planted, two-year, three-year and four-year consecutively monoculture soils, respectively. Data are means ± standard errors (one-way analysis of variance, n = 3).

Quantitative PCR confirmed the presence of *Pseudomonas* and *F. oxysporum* in all five soil samples. The density of *Pseudomonas* was significantly lower in extended monoculture soils than in control and newly planted soils while the amount of *F. oxysporum* was significantly greater in extended monoculture soil than in control and newly planted soils (Fig. 3). The result was consistent with the T-RFLP analysis (Table 1). Potential pathogens with the highest isolation frequency from extended monoculture soil and infected *R. glutinosa* were identified as *F. oxysporum* or *Fusarium* sp. (44%) (Supplementary Table S1). The ratio of *Pseudomonas* to *F. oxysporum* was significantly greater in newly planted soil than in extended monoculture soils. *In vitro* antagonism assays showed that the percent of *Pseudomonas* spp. possessing antagonistic activity against *F. oxysporum* gradually decreased with increasing years of monoculture (Fig. 4). Most of the *Pseudomonas* strains with antagonistic activity against *F. oxysporum* were the same and named as *Pseudomonas* sp. W12 in this study.

### Effects of single phenolic acids and their mixture on the growth of *Pseudomonas* sp. W12 and *F. oxysporum*

**Figure 5:**
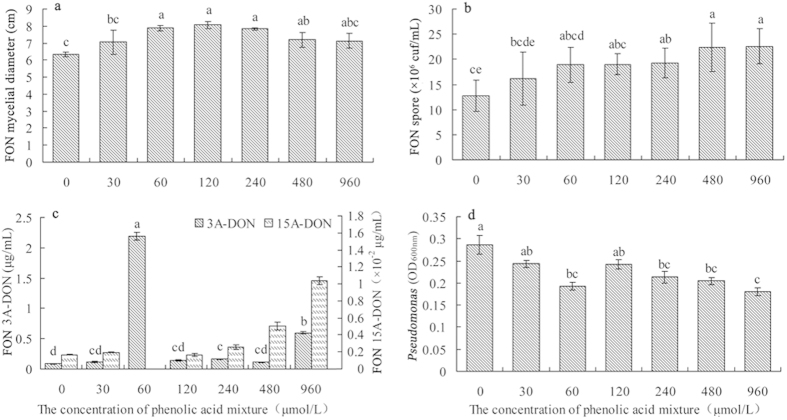
The effects of phenolic acid mixture on the mycelial growth (**a**), sporulation (**b**) and toxins production (**c**) of *F. oxysporum* (FON) and the growth of *Pseudomonas* sp. W12 (**d**).The proportion of phenolic acids was the same as the ratio detected in the rhizosphere soil of *R. glutinosa*. (**a**–**c**) Data are means ± standard errors (one-way analysis of variance, n = 3). (**d**) Data are means ± standard deviation (one-way analysis of variance, n = 4).

The effects of phenolic acid allelochemicals in root exudates on the growth of *Pseudomonas* sp. W12 and *F. oxysporum* were determined. A phenolic acid mixture at the same ratio as that detected in rhizosphere soil, significantly promoted mycelial growth of *F. oxysporum* (Fig. 5a). Among the eight phenolic compounds identified in the soil, vanillic acid and ferulic acid had the highest stimulatory effect on the growth of *F. oxysporum* (Supplementary Fig. S4a). The growth promoting effect of the mixture was greater than that of single compounds. We also found that the phenolic acid mixture significantly promoted sporulation of *F. oxysporum* and the promoting effect increased as the concentration increased (Fig. 5b). Two toxins, 3-Acetyldeoxynivalenol (3A-DON) and 15-O-Acetyl-4-deoxynivalenol (15A-DON), were detected in the fermentation liquor of *F. oxysporum*. The concentration of 3A-DON was significantly higher than that of 15A-DON toxin. Addition of the exogenous phenolic acid mixture into liquid medium promoted the production of the 3A-DON toxin. The concentration of 3A-DON increased sharply and reached the highest value at 60 μmol/L (Fig. 5c). The production of 15A-DON toxin increased with increasing concentration of the phenolic acid mixture except at the 60 μmol/L level (Fig. 5c). The phenolic acid mixture at the rhizosphere soil ratio significantly inhibited *Pseudomonas* sp. W12 growth (Fig. 5d). Vanillic acid and ferulic acid had the greatest inhibitory effect on the growth of *Pseudomonas* sp. W12 (Supplementary Fig. S4b), indicating that certain allelochemicals in root exudates can have the selective effect on rhizosphere microbes.

### High pathogenicity of *F. oxysporum* and biocontrol effects of *Pseudomonas* sp. W12

**Figure 6:**
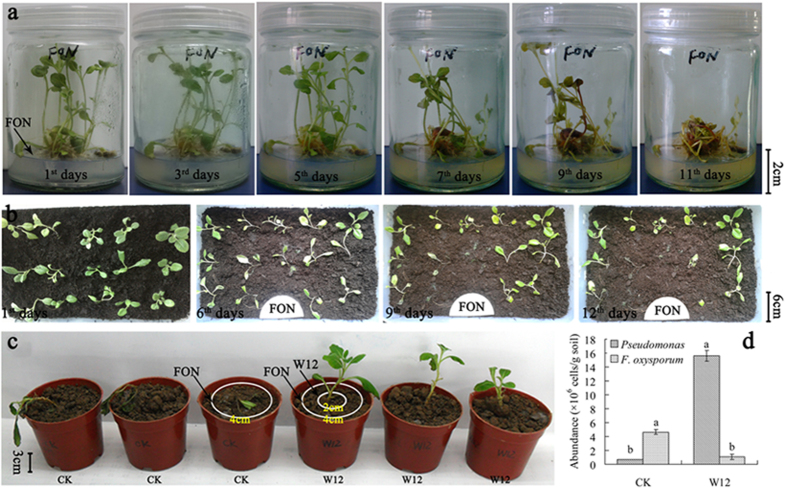
Assessment of the pathogenicity of isolated *F. oxysporum* (**a** and **b**) and the biocontrol potential of *Pseudomonas* sp. W12 (**c** and **d**). (**b**) *F. oxysporum* (FON) was inoculated in the filter paper. (**c**) *Pseudomonas* sp. W12 (W12) was inoculated around 2 cm away from the seedling root and *F. oxysporum* (FON) was inoculated around 4 cm away from the seedling root. Equal amount of LB broth medium was added in the control (CK) to replace the fermentation broth of *Pseudomonas* sp. W12. (**d**) Data are means ± standard errors (one-way analysis of variance, n = 4).

The purified isolate of *F. oxysporum* rapidly caused wilt disease on the tissue culture seedlings of *R. glutinosa*. This occurred in the tissue culture vessels under sterile conditions and also in pots with sterilized soils (Fig. 6a,b). *F. oxysporum* invades roots and colonizes xylem vessels to cause wilt disease. When the pathogenic fungus was isolated from the infected plant parts it was verified to be the same as the inoculated strain based on morphological observation and ITS region sequencing. This demonstrated the high pathogenicity of *F. oxysporum* isolate to the *R. glutinosa*.

The antagonistic effect of *Pseudomonas* sp. W12 against *F. oxysporum* was evaluated. Transplanted tissue culture seedlings of *R. glutinosa* treated with *F. oxysporum* (inoculated around 4 cm away from the seedling root) (CK) soon developed disease symptoms, withered, and died. However, using *Pseudomonas* sp. W12 (inoculated around 2 cm away from the seedling root) to inhibit *F. oxysporum* (inoculated around 4 cm away from the seedling root) (W12 treatment), the tissue culture seedling of *R. glutinosa* grew well and exhibited no disease symptoms during the entire experiment period (Fig. 6c). Quantitative PCR confirmed that the amount of *Pseudomonas* was significantly higher in the W12 treatment than in the control (CK) while the amount of *F. oxysporum* showed the opposite trend (Fig. 6d), which was consistent with the disease severity of the seedlings. These results indicate that the exogenous addition of antagonistic *Pseudomonas* can effectively decrease plant infection by *F. oxysporum*. The results also demonstrate that imbalances between these two strains (*Pseudomonas* sp. W12 and *F. oxysporum*) can lead to serious disease problems in extended monoculture. The results clearly show the biocontrol potential of the antagonistic *Pseudomonas* sp. W12.

## Discussion

The consecutive monoculture problem, also known as replanting disease, is typically manifested as a dramatic reduction in plant growth, with a shortened production life, and a decline in biomass and yield^22^. Our four-year field experiment showed the typical growth inhibition effects caused by extended *R. glutinosa* monoculture (Fig. 1). Two-year consecutive monoculture can lead to complete loss of the root tubers. More attention is being given to the biological relationships that occur between plants and microorganisms in the root zone, which are crucial for plant growth and health^8^,^23^. In this study, T-RFLP results showed significant shifts in both bacterial and fungal communities in rhizosphere after extended monoculture of *R. glutinosa* (Fig. 2). Latz *et al*.^24^ suggested that plant diversity improved protection against soil-borne pathogens by fostering antagonistic bacterial communities while plant monocultures had a negative impact on the abundance of antagonistic bacteria. Based on T-RFLP PAT assignation, we found that extended monoculture of *R. glutinosa* resulted in significant reduction of *Pseudomonas* populations (Table 1). Quantitative PCR assay confirmed the decline in *Pseudomonas* abundance (Fig. 3). Furthermore, plate counts showed that the relative abundance of *Pseudomonas* spp. with antagonistic activity against pathogenic *F. oxysporum* was significantly lower in extended monoculture soil than in newly planted soil (Fig. 4). Previous studies demonstrated that the increase of antagonistic *Pseudomonas* spp. that control specialized pathogens increased disease suppression in soils^25^,^26^. Disease-suppressive soil has been studied for take-all decline of wheat, which occurs after several consecutive years of wheat monoculture^26^. The decline of take-all disease resulted from the increase of antagonistic *Pseudomonas* populations that produce antifungal compounds such as 2,4-diacetylphloroglucinol^27^,^28^,^29^. Induction of disease suppression in soils after several years of crop monoculture has been reported for several diseases^8^. In our four-year field trial, we did not observe the development of disease suppression, perhaps due to the limited monoculture duration. However, we found the abundance of antagonistic *Pseudomonas* spp. decreased with duration of the monoculture indicating the importance of antagonistic *Pseudomonas* spp. in soil health and plant performance.

An enormous variety of potentially valuable low-molecular weight organic compounds are exuded by plant roots into the rhizosphere. These compounds in the rhizosphere have an ecological role in plant-microbe-soil interactions^30^,^31^. We identified pathogenic bacteria in the extended monoculture soil that might benefit from fresh carbon released by *R. glutinosa* roots. *F. oxysporum* or *Fusarium* sp. corresponding with T-RFs *Hinf*III 304 bp and *Taq*I 251 bp were significantly more abundant in extended monoculture soil than in newly planted soil (Table 1). *Fusarium* spp. are a well-known pathogens for many important crops. Culture-based study and quantitative PCR showed that *F. oxysporum* was a common pathogen in replanted soil and its density was significantly higher in extended monoculture soils (Fig. 3, Supplementary Table S1). Root exudates have pronounced selective effects on specific microbial populations in soil, which can deter one organism while attracting others^14^,^32^. Zhou *et al*.^33^ found *p*-coumaric acid, an autotoxin of cucumber, increased *F. oxysporum* f.sp. *cucumerinum* Owen (a soil-borne pathogen of cucumber) population densities in soil and increased the severity of Fusarium wilt under field conditions. Sugars and amino acids in root exudates can promote spore germination and mycelial growth of the soil-borne pathogen *F. oxysporum*^34^. We found that the phenolic acid mixture, at the same ratio found in the *R. glutinosa* rhizosphere, could greatly promote the mycelial growth, sporulation, and toxin production of pathogenic *F. oxysporum* (Fig. 5). However, the test mixture could greatly inhibit the growth of beneficial *Pseudomonas* sp. W12 (Fig. 5). Bais *et al*.^35^ found that rosmarinic acid, an antimicrobial compound released by *Ocimum basilicum* roots upon microbe attack, had a significant deleterious effects on *Pseudomonas aeruginosa*. *P. aeruginosa* is commonly used as a biocontrol agent for management of *Fusarium* wilt disease^36^.

**Figure 7:**
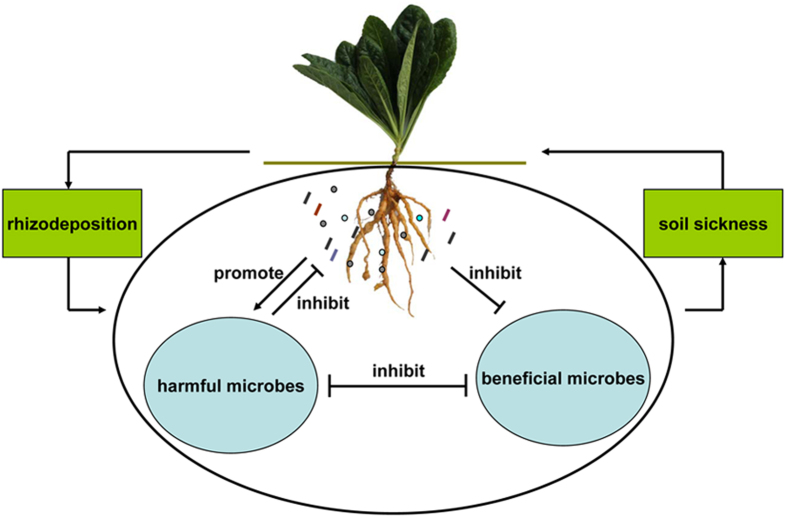
Proposed model for the underlying mechanism of consecutive monoculture problems of *R. glutinosa*. The plant in this figure was photographed and edited by Linkun Wu.

Our results indicate that the biomass decline of *R. glutinosa* under four-year extended monoculture is influenced by at least two factors: i) the decline of beneficial bacteria with antagonistic activity against pathogens in the rhizosphere and ii) an increase in disease susceptibility due to poor growth of *R. glutinosa* at a time when pathogenic microbes have became dominant (Fig. 7). This knowledge is essential in developing methods to solve consecutive monoculture problems in *R. glutinosa* and other crops. Such methods might include the use of microbial fertilizer and organic amendment application. Additional work is needed to characterize aboveground plant performance and belowground microbial diversity after a long-term monoculture of *R. glutinosa*, The mechanisms by which antagonistic microbes control disease organisms would also be a useful research area.

## Methods

### Field experiment and soil sampling

*R. glutinosa* cultivar ‘Wen 85–5’, common in the main production region, was used as the test plant material. *R. glutinosa* was typically planted on April 15 and harvested by October 30. After harvest, fields were kept fallow from October 31 to the following April 15. The experiment was conducted at Wenxian Agricultural Institute, Jiaozuo City, Henan Province (34°56′N, 112°58’E). This area is the “geo-authentic” zone for *R. glutinosa* cultivation. It has a continental monsoon climate, annual mean temperature of 14.3 °C and an annual mean precipitation of 552 mm. A field previously cultivated with wheat was used for this experiment (sandy loam soil, total nitrogen 0.48 g·kg^−1^, available nitrogen 21.34 mg·kg^−1^, total phosphorus 1.36 g·kg^−1^, available phosphorus 47.02 mg·kg^−1^, total potassium 7.23 g·kg^−1^, available potassium 251.34 mg·kg^−1^, soil organic matter 10.32 g·kg^−1^ and pH 7.52). The treatments were: i) the newly planted (NP), ii) two-year consecutive monoculture (SM), iii) three-year consecutive monoculture (TM), iv) four-year consecutive monoculture (FOM) and v) control with no *R. glutinosa* cultivation (CK) (Table 2). The above treatments were organized within a single field site with the same climatic conditions and closely adjacent to each other. Each treatment had three replicate plots and the study plots were completely randomized. Prior to planting, each replicate plot received four fertilizers: 1.6 kg N-P-K complex fertilizer, 1.25 kg (NH_4_)_2_HPO_4_, 1.6 kg Ca(H_2_PO_4_)_2_, and 0.8 kg K_2_SO_4_. All treatments were subjected to the same fertilization and field management during the entire experimental period.

On August 1, 2013 we collected soil samples from 5 random locations within each plot because of the significant difference in growth status between different treatments on this date (Fig. 1). Three plants were sampled at each location. Soil samples from all five treatments were collected at the same time. Fresh plants were carefully uprooted from the soil with a forked spade and slightly shaken to remove loosely attached soil. The rhizosphere soil that was tightly attached to roots and rhizomes was brushed off and collected. Plots without *R. glutinosa* served as a bulk soil control for contrast with rhizosphere communities under the influence of the host plant. Soil samples were sieved through 2 mm mesh to determine chemical properties (Supplementary Table S2), extraction of soil DNA, and identification of soil phenolic acids. Some soil samples were stored in 4 °C for up to a week for microbe isolation.

### The extraction and quantification analysis of phenolic acids from root exudates and rhizosphere soil

A series of tissue culture seedlings of *R. glutinosa* were incubated under sterile condition for different lengths of time. Each vessel had 30 mL of Murashige-Skoog medium (supplemented with 0.2 mg/L 6-BA, 0.2 mg/L indole-3- butyric acid and 30 g/L sucrose) and three tissue culture seedlings. The root exudates in MS medium were first extracted by using 1 mol/L NaOH. The extract was adjusted to pH 2.5 with HCl and extracted five times with an equal volume of ethyl acetate. The pooled extracts were evaporated to dryness at 35 °C and dissolved in 5 mL methanol. Quantification analysis of phenolic acids in root exudates was carried out using high performance liquid chromatography (HPLC) (Shimadzu, Japan) equipped with an ODS—C18 column (Inertsil ODS-SP, 4.6 × 250 mm, 5 μm, Japan). The mobile phase was a mixture of 28% phase A (100% methanol) and 72% phase B (2% acetic acid) maintained at a constant flow of 0.8 mL/min. The column temperature was maintained at 30 °C and detection was performed at 280 nm. Identification and quantification of phenolic acids were performed based on the retention times and the addition of pure standards to the samples. The extraction and quantification analysis of phenolic acids from rhizosphere soil was the same as that of root exudates of tissue culture seedlings.

### Terminal restriction fragment length polymorphism (T-RFLP)

We extracted total DNA from soil samples in triplicate using SoilGen DNA kit (CWBIO, Beijing, China) following the manufacturer’s instructions. Bacterial 16S rRNA gene was amplified with 6-carboxyflurescein-labeled primer 27F-FAM (5′-AGAGTTTGATCCTGGC TCAG-3′) and 1492R (5′-GGTTACCTTGTTACGACTT-3′). The fungal internal transcribed spacer (ITS) region was amplified with 6-carboxyflurescein-labeled primer ITS1F-FAM (5′-CTTGGTCATTTAGAGGAAGTAA-3′) and ITS4 (5′-TCCTCCGCTTATTGATATGC-3′). Purified bacterial 16S rRNA fragments were digested with restriction endonucleases *Msp*I, *Hae*III and *Alu*I. Purified fungal ITS fragments were digested with restriction endonucleases *Alu*I and *Hinf*I for 5 h at 37 °C, and with *Taq*I for 5 h at 65 °C. The length of terminal restriction fragments (T-RFs) was determined by the ABI 3730xl DNA sequencer (Applied Biosystems, Foster City, USA) in the GeneScan mode. Additional details on the experimental protocol of T-RFLP analysis were presented in Supplementary Material 1.

The T-RFLP Phylogenetic Assignment Tool (PAT) was used to find possible phylogenetic assignments of bacterial T-RF lengths^37^. For fungal T-RF identification, a database of ITS sequences from GenBank (3292 sequences downloaded using keyword ‘ITS1F’) and 50 fungal isolates obtained from the consecutive monoculture soil and infected *Rehmannia* plants (Supplementary Table S1) was established and analyzed for predicted T-RF lengths with the TRiFLe Program^38^. The T-RFs similar in size of ±1–2 bp were summarized to operational taxonomic units (OTUs)^39^. Only phylogenetic assignments that matched the experimental T-RF lengths for all three restriction enzymes were used in this study.

### Statistical analyses of T-RFLP data

We analyzed the T-RFLP profiles by GeneMarker Version 1.2 (SoftGenetics LLC, PA, USA) according to manufacturer’s instructions. The relative abundance of T-RFs was calculated as the peak area of the each T-RF divided by the total peak area of all T-RFs detected within a fragment length range between 35 and 700 bp.

Non-metric multidimensional scaling (NMDS) ordinations were used to examine relative similarities of microbial composition, which use an iterative algorithm to successively refine sample positions in the ordination with Kruskal’s stress value used as a measure of goodness of fit. A stress value less than 0.1 indicates an accurate and reliable ordination with a low probability of misinterpretation. NMDS is more robust method for investigating the microbial community data derived from T-RFLP profiles than principal components analysis (PCA) when examining non-linear data^40^. Analysis of similarity (ANOSIM) was used to examine the statistical significance between samples. Similarity percentage analysis (SIMPER) was used to assess the relative contribution (%) of each T-RF to the dissimilarity between samples, typically on an abundance basis^40^. The above multivariate statistical analyses as well as clustering analysis based on the T-RFLP data were performed with the PRIMER V5 software package (PRIMER-E Ltd, Plymouth, UK).

### Quantitative PCR for *Pseudomonas* and *F. oxysporum*

The quantitative PCR assay was used to quantify the genus *Pseudomonas* and *F. oxysporum*. Quantitative PCR was performed in 15 μl reaction mixture containing 7.5 μl 2 × SYBR green I SuperReal Premix (TIANGEN, Beijing, China), 0.5 μl of each primer (10 μM) and template DNA (20 ng of total soil DNA or a serial dilution of plasmid DNA for standard curves). The taxon-specific primer sets and their annealing temperatures are listed in Supplementary Table S3. Four independent quantitative PCR assays were performed for each treatment.

### Isolation and counting of *Pseudomonas* spp. with antagonistic activity

*Pseudomonas* spp. were isolated by *Pseudomonas* selective isolation agar (PSIA)^41^. Rhizosphere suspensions were prepared, serially diluted and plated onto PSIA. PSIA plates were incubated at 32 °C for 18 h. From each treatment, all colonies were purified and screened for *in vitro* antagonism towards *F. oxysporum*. For *in vitro* antagonism assays, the bacterial isolates were inoculated at the periphery of potato-dextrose-agar (PDA) plates and incubated for two days at 28 °C, after which the isolated *F. oxysporum* was transferred to the centre of the plates. After an additional three days of incubation at 28 °C, *Pseudomonas* spp. with antagonistic activity against *F. oxysporum* were recorded.

### The effects of phenolic acids on the growth of *Pseudomonas* sp. W12 and *F. oxysporum*

Based on HPLC analysis, eight phenolic acids including protocatechuic acid, phthalic acid, *p*- hydroxybenzoic acid, vanillic acid, syringic acid, vanillin, ferulic acid, benzoic acid were identified in rhizosphere soil. These standard compounds were first dissolved in small amount of methanol and then prepared with distilled water into a mixture at the same ratio as detected in rhizosphere soil of *R. glutinosa*. The isolated *F. oxysporum* was inoculated onto the center of 9 cm diam Petri dishes filled with a 10-fold dilution of soil-extract agar medium (SEM) containing different final concentrations of single phenolic acids or mixtures (30, 60, 120, 240, 480 and 960 μmol/L). The soil-extract medium added into the same amount of distilled water containing equal amount of methanol was used as the control. Each treatment had three replicates. After eight days of incubation at 28 °C in 0:24 L:D, colony diameter was measured. Sporulation of *F. oxysporum* was achieved by adding the test mixture to 8-fold dilution of potato-sugar-agar (PSA) broth medium. After seven days of incubation in a liquid shaker with 180 rpm at 28 °C, the spores were counted by hemocytometer and converted to the number of conidia in a liquid culture. Likewise, for toxin detection, the same amount of spore suspension was inoculated into a 10-fold dilution of SEM broth medium containing different concentrations of phenolic acid mixture and incubated in a liquid shaker with 180 rpm at 28 °C for ten days. At the end of the incubation period the fermentation broth was filtered through 0.22 μm filter membrane and extracted with an equal volume of ethyl acetate in a shaker with 160 rpm for 8 h. After centrifugation, the organic phase in the upper layer was vacuum-dried and dissolved in distilled water. Three toxins in the extracts including deoxynivalenol (DON), 3-Acetyldeoxynivalenol (3A-DON) and 15-O-Acetyl-4-deoxynivalenol (15A-DON) were detected using the HPLC system with an ODS—C18 column (Inertsil ODS-SP, 4.6 × 250 mm, 5 μm). The mobile phase consisted of 100% acetonitrile (A) and 0.005% phosphoric acid (B) with a gradient elution of 0 min; 5% A and 95% B → 9 min; 70% A and 30% B → 18 min; 100% A → 23 min; 100% A → 23.01 min; 5% A and 95% B → 45 min; 5% A and 95% B at a constant rate of 0.7 mL/min. The UV monitor was set to 224 nm. The column temperature was maintained at 35 °C. The toxins were identified by comparing the retention times with those of the corresponding standard compounds.

The effects of single phenolic acids and their mixture at the same ratio as detected in rhizosphere soil on the growth of isolated *Pseudomonas* sp. W12 were determined by adding the test compounds to an 8-fold dilution of LB broth medium. The LB broth medium added into the same amount of distilled water containing an equal amount of methanol was used as the control. After 8–10 h incubation in a liquid shaker at 200 rpm and 28 °C, bacterial culture density, expressed as optical density (OD) was determined at 600 nm with a plate reader (Thermo Scientific Multiskan MK3, Shanghai, China).

### Assessment of the pathogenicity of *F. oxysporum* and the biocontrol potential of *Pseudomonas* sp. W12

Tissue culture seedlings of *R. glutinosa* were incubated on specific MS medium for 45 d and then transplanted to plastic pots filled with sterilized growing medium for 14 d. The pots were placed in a growth chamber at 25 °C with a photoperiod (L:D) of 16:8. Isolated *F. oxysporum* was inoculated in a liquid shaker at 180 rpm and 28 °C at (L:D) 0:24 for 2 d. Then spore suspension was added to the tissue culture vessels under sterile conditions and the transplanted seedlings to test the effects of *F. oxysporum* on the development of *Fusarium* wilt of *R. glutinosa*. Assessment of biocontrol potential of isolated *Pseudomonas* sp. W12 was conducted by exogenous addition of *Pseudomonas* sp. W12 (inoculated 2 cm away from the seedling root) and *F. oxysporum* (inoculated 4 cm away from the seedling root). The transplanted tissue culture seedlings were only treated by *F. oxysporum* (inoculated 4 cm away from the seedling root). Equal amounts of LB broth medium were used as the controls. Each treatment had three replicates.

## Additional Information

**How to cite this article**: Wu, L. *et al*. Plant-microbe rhizosphere interactions mediated by *Rehmannia glutinosa* root exudates under consecutive monoculture. *Sci. Rep*. **5**, 15871; doi: 10.1038/srep15871 (2015).

## Supplementary Material

Supplementary Information

## Figures and Tables

**Table 1:** Top terminal restriction fragments (T-RFs) with 20% cumulative contribution to the dissimilarity between the newly planted and consecutively monocultured soils.

Discriminating T-RFs (bp)	Enzymes	Contribution (%)	Relative abundance (%)					TRFLP-PAT assignment
			CK	NP	SM	TM	FOM	
Bacteria								
37	*Hae*III	3.48	7.99	2.76	4.09	7.57	10.10	/
252	*Alu*I	2.65	12.19	10.73	12.43	14.66	15.35	/
295	*Hae*III	1.88	11.05	8.04	7.97	10.54	12.44	/
293	*Msp*I	1.54	5.74	6.52	5.54	3.99	3.35	/
486	*Msp*I	1.32	1.21	1.47	2.60	4.15	3.37	/
329	*Hae*III	1.27	3.84	3.43	2.49	0	3.51	/
327	*Hae*III	1.26	0	0	2.03	2.15	1.27	/
205	*Hae*III	1.25	2.20	4.20	2.73	2.52	1.90	/
150	*Msp*I	1.21	1.16	0.95	2.04	2.69	3.38	/
92	*Msp*I	1.18	12.63	4.57	4.56	8.19	5.70	/
249	*Alu*I	1.13	2.89	4.82	3.24	3.50	2.66	/
72	*Alu*I	1.43	0.52	2.20	0.47	0.72	0.73	*Pseudomonas* sp.
141	*Msp*I	1.08	12.84	9.01	7.31	6.96	8.37	*Burkholderia* NF100, str. AS2987
Fungi								
251	*Taq*I	2.98	4.17	0	0.39	31.80	2.35	*Fusarium oxysporum* isolate 850
428	*Alu*I	2.86	0.57	12.13	1.39	0.33	1.53	/
204	*Alu*I	2.77	0	0	0	30.57	1.46	/
361	*Hinf*III	2.52	0.70	0	0	26.21	3.03	/
304	*Hinf*III	2.38	0	0	24.87	0.74	1.93	*Fusarium oxysporum* isolate 850
357	*Taq*I	2.18	0	0	23.10	0.57	1.61	/
65	*Hinf*III	1.76	0.64	8.25	0	0.42	3.97	/
93	*Alu*I	1.7	0	0.41	18.44	0.53	2.00	/
588	*Alu*I	1.45	0.57	12.13	1.39	0.33	1.53	/

CK, NP, SM, TM and FOM represent the control, newly planted, two-year, three-year and four-year consecutive monoculture soils, respectively.

**Table 2:** Growth periods of five different treatments.

Treatments	Site code	Apr. 15, 2010- Oct. 30, 2010	Apr. 15, 2011- Oct. 30, 2011	Apr. 15, 2012- Oct. 30, 2012	Apr. 15, 2013- Oct. 30, 2013	Aug. 1, 2013
Control (unplanted) soil	CK	fallow	fallow	fallow	fallow	sampled
Newly planted soil	NP	fallow	fallow	fallow	planted	sampled
Two-year monoculture soil	SM	fallow	fallow	planted	planted	sampled
Three-year monoculture soil	TM	fallow	planted	planted	planted	sampled
Four-year monoculture soil	FOM	planted	planted	planted	planted	sampled
