# Corrigendum: Plant-microbe rhizosphere interactions mediated by *Rehmannia glutinosa* root exudates under consecutive monoculture

**DOI:** 10.1038/srep19101

**Published:** 2016-01-07

**Authors:** Linkun Wu, Juanying Wang, Weimin Huang, Hongmiao Wu, Jun Chen, Yanqiu Yang, Zhongyi Zhang, Wenxiong Lin

Scientific Reports 5: Article numnber: 15871; 10.1038/srep15871published online: 10302015; updated: 01072016

The Acknowledgements section in this Article is incomplete.

“This work was supported by grants from the National Science Foundation of China (Grant nos 81303170, U1205021 and 2012CB126309) and the Scientific Research Foundation of Graduate School of Fujian Agriculture and Forestry University (324-1122yb005).”

should read:

“This work was supported by grants from the National Science Foundation of China (Grant nos. 81303170, U1205021 and 2012CB126309), the Outstanding Youth Scientific Fund of Fujian Agriculture and Forestry University (Grant no. XJQ201501) and the Scientific Research Foundation of Graduate School of Fujian Agriculture and Forestry University (324-1122yb005).”

