# Supplementary material for: Plant-microbe rhizosphere interactions mediated by Rehmannia glutinosa root exudates under consecutive monoculture
Source: Sci Rep. 2015 Oct 30;5:15871. doi: 10.1038/srep15871 (PMC4626807; doi:10.1038/srep15871)
Supplement: Supplementary Information [file srep15871-s1.doc]

**Plant-microbe rhizosphere interactions mediated by *Rehmannia glutinosa* root exudates under consecutive monoculture**

Linkun Wu^1,2^, Juanying Wang^1,2^, Weimin Huang^1,2^, Hongmiao Wu^1,2^, Jun Chen^1,2^, Yanqiu Yang^1,2^, Zhongyi Zhang^3^ & Wenxiong Lin^1,3^

^1^Key Laboratory of Biopesticide and Chemical Biology, Ministry of Education, Fujian Agriculture and Forestry University, Fuzhou 350002, Fujian, P. R. China.

^2^College of Life Sciences, Fujian Agriculture and Forestry University, Fuzhou 350002, Fujian, P. R. China.

^3^Fujian Provincial Key Laboratory of Agroecological Processing and Safety Monitoring, College of Life Sciences, Fujian Agriculture and Forestry University, Fuzhou 35002, Fujian, P. R. China.

Correspondence and requests for materials should be addressed to W.X.L. (email: lwx@fafu.edu.cn).

**Additional information**

**Supplementary Material 1:** The experimental protocol for terminal restriction fragment length polymorphism (T-RFLP) analysis

**Supplementary Figure S1 | Identification and quantification of phenolic acid compounds in root exudates under sterile condition (a) and in rhizosphere soil (b).** **a**: Legend indicates the different lengths of growth time for tissue culture seedlings. **b**: Data are means ± standard deviation (one-way analysis of variance, n = 3). CK, NP, SM, TM and FOM represent the control, newly planted, two-year, three-year and four-year consecutively monocultured soils, respectively.

**Supplementary Figure S2 | T-RFLP profiles of bacterial 16S rRNA genes amplified from the control (CK), newly planted (NP), two-year (SM), three-year (TM), and four-year (FOM) monoculture soils for each restriction enzyme (*Msp*I, *Hae*III and *Alu*I).**

**Supplementary Figure S3 | T-RFLP profiles of fungal ITS rRNA genes amplified from the control (CK), newly planted (NP), two-year (SM), three-year (TM), and four-year (FOM) monoculture soils for each restriction enzyme (*Alu*I, *Hinf*I and *Taq*I).**

**Supplementary Figure S4 | Effects of single phenolic acids on the growth of *F. oxysporum* (FON) (a) and *Pseudomonas* sp. W12 (b). a**: Data are means ± standard errors (one-way analysis of variance, n = 3). **b**: Data are means ± standard deviation (one-way analysis of variance, n = 4).

**Supplementary Table S1 | Nucleotide database analysis of fungal isolates from consecutive monoculture soil and infected *Rehmannia* plants (top BLAST hit for ITS region).**

**Supplementary Table S2 | Chemical properties of soils from five different treatment plots.**

**Supplementary Table S3 | Taxon-specific primer sets and their annealing temperatures for quantitative PCR.**

**Supplementary material 1:** The experimental protocol for terminal restriction fragment length polymorphism (T-RFLP) analysis

We extracted total DNA from the collected soil samples in triplicate using SoilGen DNA kit (CWBIO, Beijing, China) following the manufacturer’s instructions. Bacterial 16S rRNA gene was amplified with 6-carboxyflurescein-labeled primer 27F-FAM (5’-AGAGTTTGATCCTGGCTCAG-3’) and 1492R (5’-GGTTACCTTGTTACGACTT-3’). Fungal internal transcribed spacer (ITS) region was amplified with 6-carboxyflurescein-labeled primer ITS1F-FAM (5’-CTTGGTCATTTAGAGGAAGTAA-3’) and ITS4 (5’-TCCTCCGCTTATTGATATGC-3’). The reaction mixture consisted of 25 μl Taq PCR Master Mix (2×) (Sangon, Shanghai, China), 1.5 μl of each primer (10 μM) and 40 ng template DNA in final volume of 50 μl. Thermocycling for 16s rRNA gene amplification consisted of an initial denaturation at 94 °C for 5 min, followed by 35 cycles of denaturation at 94°C for 60 sec, annealing at 55°C for 45 sec, extension at 72 °C for 90 sec and a final extension for 10 min at 72°C. Thermocycling for ITS region amplification consisted of an initial denaturation at 94 °C for 5 min, followed by 35 cycles of denaturation at 94°C for 45 sec, annealing at 51°C for 45 sec, extension at 72 °C for 60 sec and a final extension for 10 min at 72°C. The PCR reaction was carried out in quadruplicate for each replicate. The PCR product was subjected to 1% agarose gel electrophoresis and purified by using Universal DNA Purification Kit (TIANGEN, Beijing, China) prior to restriction digestion. The DNA was quantified by determining absorption of samples at 260 nm.

Purified bacterial 16S rRNA fragments were digested with restriction endonucleases *Msp*I, *Hae*III and *Alu*I for 5 h at 37 °C. The restriction digestion mixture for *Msp*I consisted 10 μl purified fragments (0.6-0.8 μg), 2 μl 10×T buffer, 2 μl of 0.1% BSA (TAKARA BIO, Otsu, Japan) and 1 μl *Msp*I (10 U) in final volume of 20 μl. The restriction digestion mixture for *Hae*III or *Alu*I consisted of 10 μl purified fragments (0.6-0.8 μg), 2 μl 10×L buffer and 1 μl *Hae*III or *Alu*I (10 U) in final volume of 20 μl.

Purified fungal ITS fragments were digested with restriction endonucleases *Alu*I and *Hinf*I for 5 h at 37 °C, and with *Taq*I for 5 h at 65 °C. Purified DNA (10 μl, 0.6-0.8 μg) was digested with 2 μl buffer (10×L for *Alu*I and 10×H for *Hinf*I) and 1 μl *Alu*I or *Hinf*I (10 U) in a 20 μl reaction volume. The restriction digestion mixture for *Taq*I consisted of 10 μl purified DNA (0.6-0.8 μg), 2 μl 10× *Taq*I buffer, 2 μl of 0.1% BSA (TAKARA BIO, Otsu, Japan) and 1 μl *Taq*I (10 U).

The digested PCR products (1 μl) were mixed with 9.9 μl of deionized formamide and 0.1 μl of GeneScan LIZ-500 size standard (Applied Biosystems, Foster City, USA). This mixture was denatured at 98°C for 5 min and then immediately stored on ice prior to electrophoresis. The length of terminal restriction fragments (T-RFs) was determined by the ABI 3730xl DNA sequencer (Applied Biosystems, Foster City, USA) in the GeneScan mode.


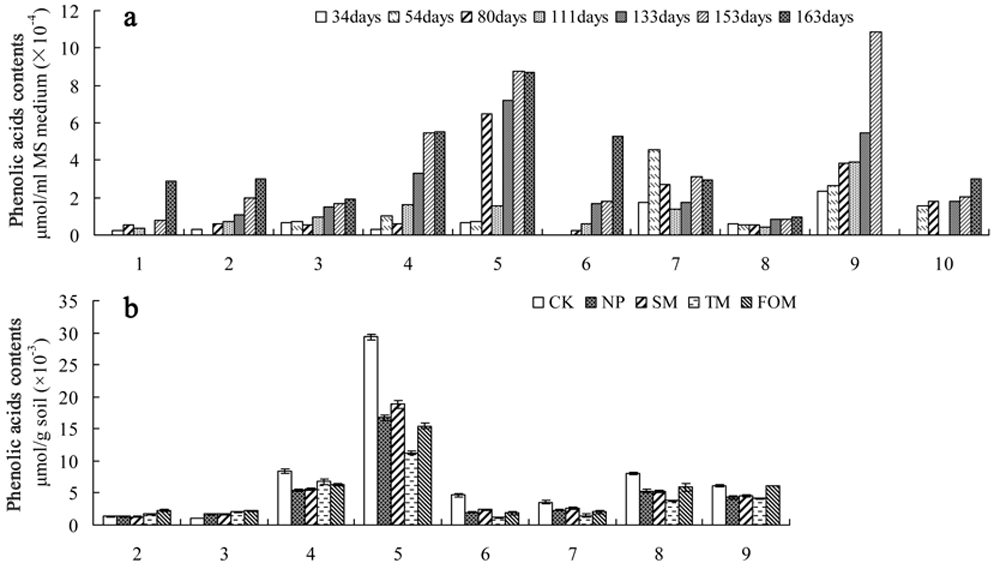


**Supplementary Figure S1 | Identification and quantification of phenolic acid compounds in root exudates under sterile condition (a) and in rhizosphere soil (b).** **a**: Legend indicates the different lengths of growth time for tissue culture seedlings. **b**: Data are means ± standard deviation (one-way analysis of variance, n = 3). CK, NP, SM, TM and FOM represent the control, newly planted, two-year, three-year and four-year consecutively monocultured soils, respectively.


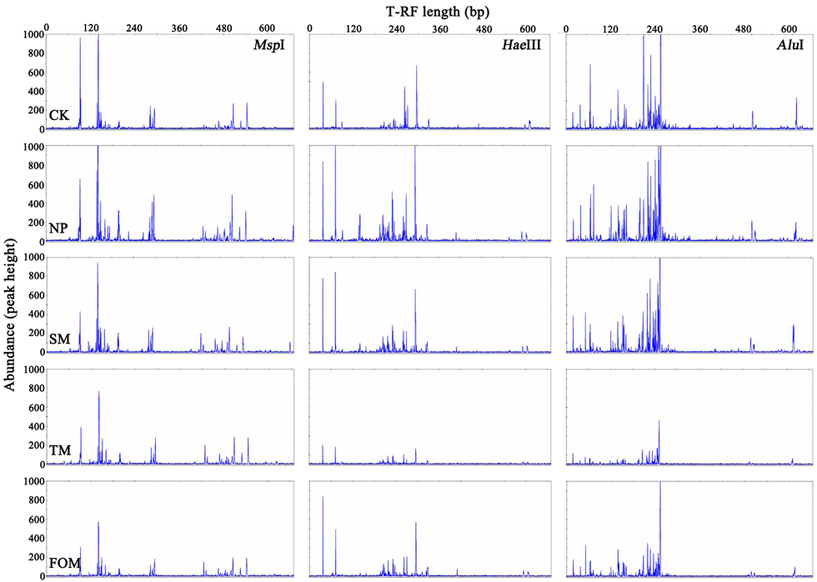


**Supplementary Figure S2 | T-RFLP profiles of bacterial 16S rRNA genes amplified from the control (CK), newly planted (NP), two-year (SM), three-year (TM), and four-year (FOM) monoculture soils for each restriction enzyme (*Msp*I, *Hae*III and *Alu*I).**


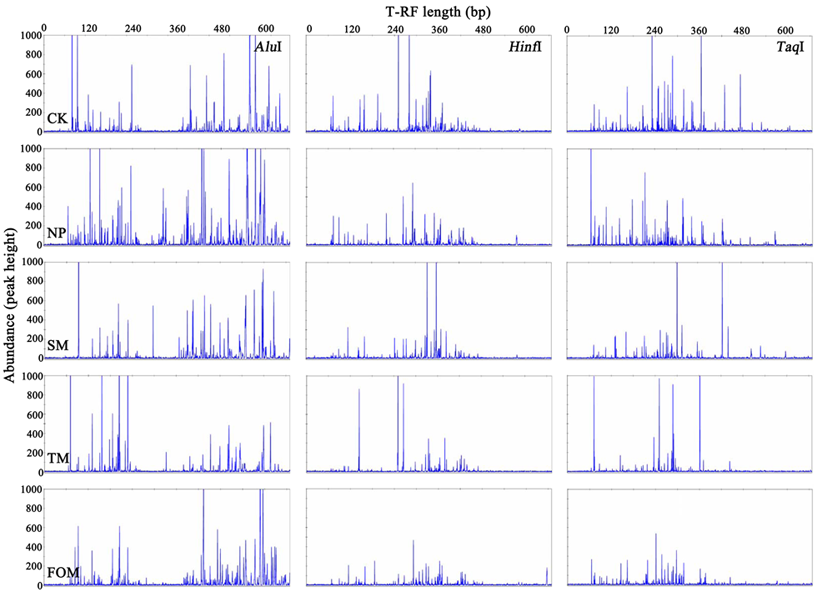


**Supplementary Figure S3 | T-RFLP profiles of fungal ITS rRNA genes amplified from the control (CK), newly planted (NP), two-year (SM), three-year (TM), and four-year (FOM) monoculture soils for each restriction enzyme (*Alu*I, *Hinf*I and *Taq*I).**


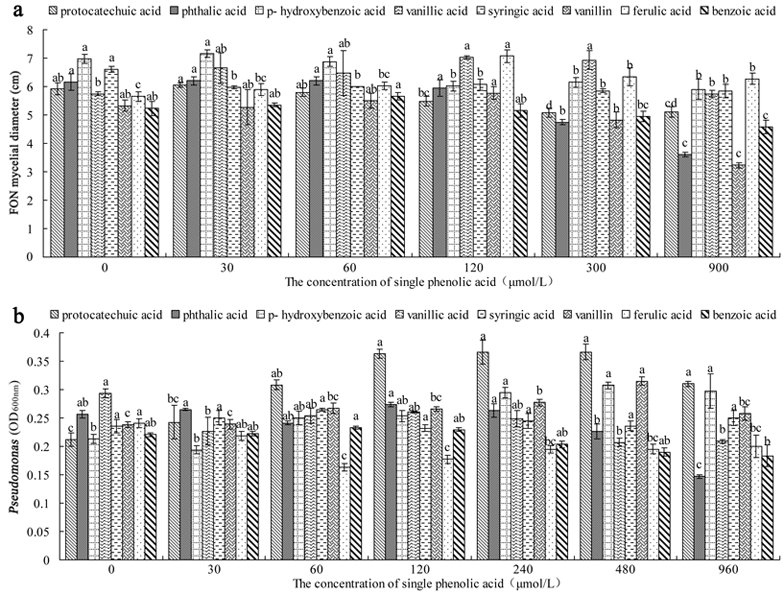


**Supplementary Figure S4 | Effects of single phenolic acids on the growth of *F. oxysporum* (FON) (a) and *Pseudomonas* sp. W12 (b). a**: Data are means ± standard errors (one-way analysis of variance, n = 3). **b**: Data are means ± standard deviation (one-way analysis of variance, n = 4).

**Supplementary Table S1 | Nucleotide database analysis of fungal isolates from consecutive monoculture soil and infected *Rehmannia* plants (top BLAST hit for ITS region).**

| **Isolate code** | **Genbank ID** | **Genbank description** | **Blast**  **E-value** | **%identity** | ***in silico* T-RFs** | | | **Phylum** |
| --- | --- | --- | --- | --- | --- | --- | --- | --- |
|  |  |  |  |  | AluI | HinfI | TaqI |  |
| CMS8 | [FJ210507.1](http://www.ncbi.nlm.nih.gov/nucleotide/226442101?report=genbank&log$=nucltop&blast_rank=1&RID=SPDMFUPH01R) | *[Mucor](http://blast.ncbi.nlm.nih.gov/Blast.cgi#alnHdr_226442101)* sp. JJP-2009a | 0.0 | 100% | 196 | 183 | 310 | [*Zygomycota*](http://en.wikipedia.org/wiki/Zygomycota) |
| CMS20 | [FJ011538.1](http://www.ncbi.nlm.nih.gov/nucleotide/198401226?report=genbank&log$=nucltop&blast_rank=1&RID=SPF260ZF01R) | *[Aspergillus terreus](http://blast.ncbi.nlm.nih.gov/Blast.cgi#alnHdr_198401226)* isolate SUMS0191 | 0.0 | 99% | 219 | 163 | 288 | [*Ascomycota*](http://en.wikipedia.org/wiki/Ascomycota) |
| CMS9 | [AB026011.1](http://www.ncbi.nlm.nih.gov/nucleotide/7415586?report=genbank&log$=nucltop&blast_rank=1&RID=SPDTHT7201R) | *Rhodotorula* sp. SY-101 | 0.0 | 100% | 452 | 97 | 254 | [*Basidiomycota*](http://en.wikipedia.org/wiki/Basidiomycota) |
| CMS3 | JN232136.1 | *Fusarium oxysporum* isolate 850 | 0.0 | 99% | 469 | 304 | 251 | [*Ascomycota*](http://en.wikipedia.org/wiki/Ascomycota) |
| CMS24 | HQ649835.1 | *[Fusarium](http://blast.ncbi.nlm.nih.gov/Blast.cgi#alnHdr_317184078)* sp. r104 | 3e-88 | 76% | 469 | 192 | 188 | [*Ascomycota*](http://en.wikipedia.org/wiki/Ascomycota) |
| CMS1 | [JQ775558.1](http://www.ncbi.nlm.nih.gov/nucleotide/383930963?report=genbank&log$=nucltop&blast_rank=2&RID=SPBBYXH8014) | *[Fusarium](http://blast.ncbi.nlm.nih.gov/Blast.cgi#alnHdr_383930963)* sp. P62 | 0.0 | 99% | 471 | 305 | 252 | [*Ascomycota*](http://en.wikipedia.org/wiki/Ascomycota) |
| CMS2 | [HQ649835.1](http://www.ncbi.nlm.nih.gov/nucleotide/317184078?report=genbank&log$=nucltop&blast_rank=1&RID=SPCXYFM401R) | *[Fusarium](http://blast.ncbi.nlm.nih.gov/Blast.cgi#alnHdr_317184078)* sp. r104 | 0.0 | 99% | 471 | 305 | 252 | [*Ascomycota*](http://en.wikipedia.org/wiki/Ascomycota) |
| CMS6 | [HQ649839.1](http://www.ncbi.nlm.nih.gov/nucleotide/317184082?report=genbank&log$=nucltop&blast_rank=1&RID=SPBYZVME01R) | *[Fusarium](http://blast.ncbi.nlm.nih.gov/Blast.cgi#alnHdr_317184082)* sp. r323 | 0.0 | 100% | 471 | 305 | 252 | [*Ascomycota*](http://en.wikipedia.org/wiki/Ascomycota) |
| CMS12 | [JX421732.1](http://www.ncbi.nlm.nih.gov/nucleotide/402797487?report=genbank&log$=nucltop&blast_rank=1&RID=SPE1UDMM01R) | *[Aspergillus fumigatus](http://blast.ncbi.nlm.nih.gov/Blast.cgi#alnHdr_402797487)* strain ASR_H32_ | 0.0 | 100% | 593 | 221 | 224 | [*Ascomycota*](http://en.wikipedia.org/wiki/Ascomycota) |
| CMS16 | [JX421732.1](http://www.ncbi.nlm.nih.gov/nucleotide/402797487?report=genbank&log$=nucltop&blast_rank=1&RID=SPE1UDMM01R) | *[Aspergillus fumigatus](http://blast.ncbi.nlm.nih.gov/Blast.cgi#alnHdr_402797487)* strain ASR_H32 | 0.0 | 100% |  | 104 | 143 | [*Ascomycota*](http://en.wikipedia.org/wiki/Ascomycota) |
| CMS11 | [EU645733.1](http://www.ncbi.nlm.nih.gov/nucleotide/194371458?report=genbank&log$=nucltop&blast_rank=1&RID=SPDXNNWD014) | *[Aspergillus aculeatus](http://blast.ncbi.nlm.nih.gov/Blast.cgi#alnHdr_194371458)* strain JO6 | 0.0 | 100% |  | 221 | 224 | [*Ascomycota*](http://en.wikipedia.org/wiki/Ascomycota) |
| CMS21 | [KC139307.1](http://www.ncbi.nlm.nih.gov/nucleotide/444436328?report=genbank&log$=nucltop&blast_rank=1&RID=SPF50TB301R) | *[Trichoderma harzianum](http://blast.ncbi.nlm.nih.gov/Blast.cgi#alnHdr_444436328)* isolate A14 | 0.0 | 100% |  | 264 | 256 | [*Ascomycota*](http://en.wikipedia.org/wiki/Ascomycota) |
| CMS22 | [KC139307.1](http://www.ncbi.nlm.nih.gov/nucleotide/444436328?report=genbank&log$=nucltop&blast_rank=1&RID=SPF50TB301R) | *[Trichoderma harzianum](http://blast.ncbi.nlm.nih.gov/Blast.cgi#alnHdr_444436328)* isolate A14 | 0.0 | 100% |  | 264 | 256 | [*Ascomycota*](http://en.wikipedia.org/wiki/Ascomycota) |
| CMS23 | [HQ259319.1](http://www.ncbi.nlm.nih.gov/nucleotide/309261235?report=genbank&log$=nucltop&blast_rank=1&RID=SPF9DUW501R) | *[Hypocrea lixii](http://blast.ncbi.nlm.nih.gov/Blast.cgi#alnHdr_309261235)* isolate HA1302 | 0.0 | 100% |  | 264 | 256 | [*Ascomycota*](http://en.wikipedia.org/wiki/Ascomycota) |
| CMS25 | [KC139307.1](http://www.ncbi.nlm.nih.gov/nucleotide/444436328?report=genbank&log$=nucltop&blast_rank=1&RID=SPF50TB301R) | *[Trichoderma harzianum](http://blast.ncbi.nlm.nih.gov/Blast.cgi#alnHdr_444436328)* isolate A14 | 0.0 | 98% |  | 264 | 256 | [*Ascomycota*](http://en.wikipedia.org/wiki/Ascomycota) |
| CMS4 | [KC339769.1](http://www.ncbi.nlm.nih.gov/nucleotide/444891775?report=genbank&log$=nucltop&blast_rank=1&RID=SPD2JH6T01R) | *[Fusarium oxysporum](http://blast.ncbi.nlm.nih.gov/Blast.cgi#alnHdr_444891775)* isolate CNU081064 | 0.0 | 100% |  | 302 | 249 | [*Ascomycota*](http://en.wikipedia.org/wiki/Ascomycota) |
| CMS5 | [KC339767.1](http://www.ncbi.nlm.nih.gov/nucleotide/444891773?report=genbank&log$=nucltop&blast_rank=1&RID=SPC6YSEU01R) | *[Fusarium oxysporum](http://blast.ncbi.nlm.nih.gov/Blast.cgi#alnHdr_444891773)* isolate CNU081050 | 0.0 | 99% |  | 302 | 249 | [*Ascomycota*](http://en.wikipedia.org/wiki/Ascomycota) |
| CMS7 | [AY928417.1](http://www.ncbi.nlm.nih.gov/nucleotide/60593126?report=genbank&log$=nucltop&blast_rank=1&RID=SPD870JM01R) | *[Fusarium oxysporum](http://blast.ncbi.nlm.nih.gov/Blast.cgi#alnHdr_60593126)* isolate FO-10 | 0.0 | 100% |  | 302 | 249 | [*Ascomycota*](http://en.wikipedia.org/wiki/Ascomycota) |
| CMS17 | [FJ487932.1](http://www.ncbi.nlm.nih.gov/nucleotide/218938081?report=genbank&log$=nucltop&blast_rank=2&RID=SPENBGVG01R) | *[Aspergillus flavus](http://blast.ncbi.nlm.nih.gov/Blast.cgi#alnHdr_218938081)* strain ZJ4-A | 0.0 | 99% |  | 336 | 283 | [*Ascomycota*](http://en.wikipedia.org/wiki/Ascomycota) |
| CMS18 | [JX157882.1](http://www.ncbi.nlm.nih.gov/nucleotide/401667346?report=genbank&log$=nucltop&blast_rank=1&RID=SPDVPJTE01R) | *[Aspergillus flavus](http://blast.ncbi.nlm.nih.gov/Blast.cgi#alnHdr_401667346)* | 0.0 | 100% |  | 336 | 283 | [*Ascomycota*](http://en.wikipedia.org/wiki/Ascomycota) |
| CMS19 | [JX157882.1](http://www.ncbi.nlm.nih.gov/nucleotide/401667346?report=genbank&log$=nucltop&blast_rank=1&RID=SPDVPJTE01R) | *[Aspergillus flavus](http://blast.ncbi.nlm.nih.gov/Blast.cgi#alnHdr_401667346)* | 0.0 | 100% |  | 336 | 283 | [*Ascomycota*](http://en.wikipedia.org/wiki/Ascomycota) |
| CMS10 | [JX157882.1](http://www.ncbi.nlm.nih.gov/nucleotide/401667346?report=genbank&log$=nucltop&blast_rank=1&RID=SPDVPJTE01R) | *[Aspergillus flavus](http://blast.ncbi.nlm.nih.gov/Blast.cgi#alnHdr_401667346)* | 0.0 | 100% |  | 336 | 283 | [*Ascomycota*](http://en.wikipedia.org/wiki/Ascomycota) |
| CMS14 | [JQ899451.1](http://www.ncbi.nlm.nih.gov/nucleotide/440496251?report=genbank&log$=nucltop&blast_rank=1&RID=SPEF5R1H01R) | *[Aspergillus flavus](http://blast.ncbi.nlm.nih.gov/Blast.cgi#alnHdr_440496251)* strain SSM8 | 0.0 | 98% |  | 336 | 283 | [*Ascomycota*](http://en.wikipedia.org/wiki/Ascomycota) |
| CMS15 | [KC119200.1](http://www.ncbi.nlm.nih.gov/nucleotide/442571801?report=genbank&log$=nucltop&blast_rank=1&RID=SPEH5YAD01R) | *[Aspergillus fumigatus](http://blast.ncbi.nlm.nih.gov/Blast.cgi#alnHdr_442571801)* strain KARVS04 | 0.0 | 100% |  | 339 | 143 | [*Ascomycota*](http://en.wikipedia.org/wiki/Ascomycota) |
| CMS13 | [JX421732.1](http://www.ncbi.nlm.nih.gov/nucleotide/402797487?report=genbank&log$=nucltop&blast_rank=1&RID=SPE1UDMM01R) | *[Aspergillus fumigatus](http://blast.ncbi.nlm.nih.gov/Blast.cgi#alnHdr_402797487)* strain ASR_H32 | 0.0 | 99% |  | 339 | 143 | [*Ascomycota*](http://en.wikipedia.org/wiki/Ascomycota) |
| PRP14 | [HM641688.1](http://www.ncbi.nlm.nih.gov/nucleotide/305958932?report=genbank&log$=nucltop&blast_rank=1&RID=SPDH4TV501R) | *Mucor circinelloides* f. circinelloides | 0.0 | 99% | 196 | 183 | 310 | [*Zygomycota*](http://en.wikipedia.org/wiki/Zygomycota) |
| PRP11 | JQ775568.1 | *[Rhizopus](http://blast.ncbi.nlm.nih.gov/Blast.cgi#alnHdr_383930973)* sp. F36 | 0.0 | 99% | 369 | 146 | 185 | [*Zygomycota*](http://en.wikipedia.org/wiki/Zygomycota) |
| PRP12 | JQ775568.1 | *[Rhizopus](http://blast.ncbi.nlm.nih.gov/Blast.cgi#alnHdr_383930973)* sp. F36 | 0.0 | 99% | 369 | 146 | 185 | [*Zygomycota*](http://en.wikipedia.org/wiki/Zygomycota) |
| PRP13 | JQ775568.1 | *[Rhizopus](http://blast.ncbi.nlm.nih.gov/Blast.cgi#alnHdr_383930973)* sp. F36 | 0.0 | 99% | 369 | 146 | 185 | [*Zygomycota*](http://en.wikipedia.org/wiki/Zygomycota) |
| PRP6 | [JX914477.1](http://www.ncbi.nlm.nih.gov/nucleotide/432140625?report=genbank&log$=nucltop&blast_rank=1&RID=SPC4M64801R) | *[Fusarium](http://blast.ncbi.nlm.nih.gov/Blast.cgi#alnHdr_432140625)* sp. TC1-6 | 0.0 | 100% | 385 | 303 | 250 | [*Ascomycota*](http://en.wikipedia.org/wiki/Ascomycota) |
| PRP2 | [JN232163.1](http://www.ncbi.nlm.nih.gov/nucleotide/359730912?report=genbank&log$=nucltop&blast_rank=1&RID=SPBGB3WP01R) | *[Fusarium oxysporum](http://blast.ncbi.nlm.nih.gov/Blast.cgi#alnHdr_359730912)* isolate 281 | 0.0 | 100% | 389 | 302 | 249 | [*Ascomycota*](http://en.wikipedia.org/wiki/Ascomycota) |
| PRP5 | [JN232163.1](http://www.ncbi.nlm.nih.gov/nucleotide/359730912?report=genbank&log$=nucltop&blast_rank=1&RID=SPBGB3WP01R) | *[Fusarium oxysporum](http://blast.ncbi.nlm.nih.gov/Blast.cgi#alnHdr_359730912)* isolate 281 | 0.0 | 100% | 389 | 302 | 249 | [*Ascomycota*](http://en.wikipedia.org/wiki/Ascomycota) |
| PRP22 | [JF440593.1](http://www.ncbi.nlm.nih.gov/nucleotide/328942621?report=genbank&log$=nucltop&blast_rank=1&RID=SPFUCGRM01R) | *[Fusarium oxysporum](http://blast.ncbi.nlm.nih.gov/Blast.cgi#alnHdr_328942621)* | 0.0 | 100% | 389 | 302 | 249 | [*Ascomycota*](http://en.wikipedia.org/wiki/Ascomycota) |
| PRP4 | [HQ649839.1](http://www.ncbi.nlm.nih.gov/nucleotide/317184082?report=genbank&log$=nucltop&blast_rank=1&RID=SPBYZVME01R) | *[Fusarium](http://blast.ncbi.nlm.nih.gov/Blast.cgi#alnHdr_317184082)* sp. r323 | 0.0 | 98% | 471 | 305 | 252 | [*Ascomycota*](http://en.wikipedia.org/wiki/Ascomycota) |
| PRP10 | [HQ649839.1](http://www.ncbi.nlm.nih.gov/nucleotide/317184082?report=genbank&log$=nucltop&blast_rank=1&RID=SPBYZVME01R) | *[Fusarium](http://blast.ncbi.nlm.nih.gov/Blast.cgi#alnHdr_317184082)* sp. r323 | 0.0 | 100% | 471 | 305 | 252 | [*Ascomycota*](http://en.wikipedia.org/wiki/Ascomycota) |
| PRP15 | [JQ775558.1](http://www.ncbi.nlm.nih.gov/nucleotide/383930963?report=genbank&log$=nucltop&blast_rank=1&RID=SPDRC48H01R) | *[Fusarium](http://blast.ncbi.nlm.nih.gov/Blast.cgi#alnHdr_383930963)* sp. P62 | 0.0 | 100% | 471 | 305 | 252 | [*Ascomycota*](http://en.wikipedia.org/wiki/Ascomycota) |
| PRP23 | [JX179228.1](http://www.ncbi.nlm.nih.gov/nucleotide/403492124?report=genbank&log$=nucltop&blast_rank=1&RID=SPFP10WS01R) | *[Hypocreales](http://blast.ncbi.nlm.nih.gov/Blast.cgi#alnHdr_403492124)* sp. DZY07 | 0.0 | 100% | 471 | 305 | 252 | [*Ascomycota*](http://en.wikipedia.org/wiki/Ascomycota) |
| PRP24 | [HQ649839.1](http://www.ncbi.nlm.nih.gov/nucleotide/317184082?report=genbank&log$=nucltop&blast_rank=1&RID=SPBYZVME01R) | *[Fusarium](http://blast.ncbi.nlm.nih.gov/Blast.cgi#alnHdr_317184082)* sp. r323 | 0.0 | 98% | 471 | 305 | 252 | [*Ascomycota*](http://en.wikipedia.org/wiki/Ascomycota) |
| PRP25 | [HQ649839.1](http://www.ncbi.nlm.nih.gov/nucleotide/317184082?report=genbank&log$=nucltop&blast_rank=1&RID=SPBYZVME01R) | *[Fusarium](http://blast.ncbi.nlm.nih.gov/Blast.cgi#alnHdr_317184082)* sp. r323 | 0.0 | 98% | 471 | 305 | 252 | [*Ascomycota*](http://en.wikipedia.org/wiki/Ascomycota) |
| PRP21 | [JX179228.1](http://www.ncbi.nlm.nih.gov/nucleotide/403492124?report=genbank&log$=nucltop&blast_rank=1&RID=SPFP10WS01R) | *[Hypocreales](http://blast.ncbi.nlm.nih.gov/Blast.cgi#alnHdr_403492124)* sp. DZY07 | 0.0 | 100% | 472 | 306 | 253 | [*Ascomycota*](http://en.wikipedia.org/wiki/Ascomycota) |
| PRP3 | [KC339767.1](http://www.ncbi.nlm.nih.gov/nucleotide/444891773?report=genbank&log$=nucltop&blast_rank=1&RID=SPBVPGHR01R) | *[Fusarium oxysporum](http://blast.ncbi.nlm.nih.gov/Blast.cgi#alnHdr_444891773)* isolate CNU081050 | 0.0 | 100% |  | 302 | 249 | [*Ascomycota*](http://en.wikipedia.org/wiki/Ascomycota) |
| PRP7 | [KC339767.1](http://www.ncbi.nlm.nih.gov/nucleotide/444891773?report=genbank&log$=nucltop&blast_rank=1&RID=SPC6YSEU01R) | *[Fusarium oxysporum](http://blast.ncbi.nlm.nih.gov/Blast.cgi#alnHdr_444891773)* isolate CNU081050 | 0.0 | 99% |  | 302 | 249 | [*Ascomycota*](http://en.wikipedia.org/wiki/Ascomycota) |
| PRP8 | [KC339767.1](http://www.ncbi.nlm.nih.gov/nucleotide/444891773?report=genbank&log$=nucltop&blast_rank=1&RID=SPC6YSEU01R) | *[Fusarium oxysporum](http://blast.ncbi.nlm.nih.gov/Blast.cgi#alnHdr_444891773)* isolate CNU081050 | 0.0 | 99% |  | 302 | 249 | [*Ascomycota*](http://en.wikipedia.org/wiki/Ascomycota) |
| PRP9 | [AY928417.1](http://www.ncbi.nlm.nih.gov/nucleotide/60593126?report=genbank&log$=nucltop&blast_rank=1&RID=SPCAJGSB01R) | *[Fusarium oxysporum](http://blast.ncbi.nlm.nih.gov/Blast.cgi#alnHdr_60593126)* isolate FO-10 | 0.0 | 99% |  | 302 | 249 | [*Ascomycota*](http://en.wikipedia.org/wiki/Ascomycota) |
| PRP1 | [AY928415.1](http://www.ncbi.nlm.nih.gov/nucleotide/60593124?report=genbank&log$=nucltop&blast_rank=1&RID=SPB8RSEM01R) | *[Fusarium oxysporum](http://blast.ncbi.nlm.nih.gov/Blast.cgi#alnHdr_60593124)* isolate FO-08 | 0.0 | 100% |  | 303 | 250 | [*Ascomycota*](http://en.wikipedia.org/wiki/Ascomycota) |
| PRP18 | [FJ487932.1](http://www.ncbi.nlm.nih.gov/nucleotide/218938081?report=genbank&log$=nucltop&blast_rank=2&RID=SPENBGVG01R) | *[Aspergillus flavus](http://blast.ncbi.nlm.nih.gov/Blast.cgi#alnHdr_218938081)* strain ZJ4-A | 0.0 | 100% |  | 336 | 283 | [*Ascomycota*](http://en.wikipedia.org/wiki/Ascomycota) |
| PRP19 | [FJ487932.1](http://www.ncbi.nlm.nih.gov/nucleotide/218938081?report=genbank&log$=nucltop&blast_rank=2&RID=SPENBGVG01R) | *[Aspergillus flavus](http://blast.ncbi.nlm.nih.gov/Blast.cgi#alnHdr_218938081)* strain ZJ4-A | 0.0 | 100% |  | 336 | 283 | [*Ascomycota*](http://en.wikipedia.org/wiki/Ascomycota) |
| PRP20 | [JQ899451.1](http://www.ncbi.nlm.nih.gov/nucleotide/440496251?report=genbank&log$=nucltop&blast_rank=1&RID=SPEWY75B01R) | *[Aspergillus flavus](http://blast.ncbi.nlm.nih.gov/Blast.cgi#alnHdr_440496251)* strain SSM8 | 0.0 | 97% |  | 336 | 283 | [*Ascomycota*](http://en.wikipedia.org/wiki/Ascomycota) |
| PRP16 | [JX157882.1](http://www.ncbi.nlm.nih.gov/nucleotide/401667346?report=genbank&log$=nucltop&blast_rank=1&RID=SPDVPJTE01R) | *[Aspergillus flavus](http://blast.ncbi.nlm.nih.gov/Blast.cgi#alnHdr_401667346)* | 0.0 | 100% |  | 336 | 283 | [*Ascomycota*](http://en.wikipedia.org/wiki/Ascomycota) |
| PRP17 | [FJ878681.1](http://www.ncbi.nlm.nih.gov/nucleotide/237626042?report=genbank&log$=nucltop&blast_rank=1&RID=SPECY0JS01R) | *[Aspergillus flavus](http://blast.ncbi.nlm.nih.gov/Blast.cgi#alnHdr_237626042)* isolate UOA/HCPF 5774 | 0.0 | 100% |  | 336 | 283 | [*Ascomycota*](http://en.wikipedia.org/wiki/Ascomycota) |

CMS represents the fungi isolated from the consecutively monocultured soil. PRP represents the fungi isolated from the pathogenic rehmannia plants.

**Supplementary Table S2 |** Chemical properties of soils from five different treatment plots.

| Treatment | SOM  (g/kg) | TN  (g/kg) | AN  (mg/kg) | TP  (g/kg) | AP  (mg/kg) | TK  (g/kg) | AK  (mg/kg) | pH |
| --- | --- | --- | --- | --- | --- | --- | --- | --- |
| Control (unplanted) soil | 8.76b | 0.44c | 10.17b | 2.33a | 48.34b | 8.00a | 221.33b | 7.88a |
| Newly planted soil | 12.86a | 0.49b | 22.52a | 1.69b | 59.27a | 7.42a | 209.10b | 7.49b |
| Two-year monoculture soil | 13.58a | 0.57a | 22.85a | 1.21e | 64.17a | 8.23a | 360.91a | 7.45b |
| Three-year monoculture soil | 11.55a | 0.54a | 22.46a | 1.40d | 59.51a | 7.21a | 334.80a | 7.32c |
| Four-year monoculture soil | 11.36a | 0.55a | 22.37a | 1.54c | 61.83a | 7.57a | 326.51a | 7.31c |

SOM, soil organic matter; TN, total nitrogen; AN, available nitrogen; TP, total phosphorus; AP, available phosphorus; TK, total potassium; AK, available potassium. Different letters in columns show significant differences determined by Tukey's test (*P* ≤ 0.05, n=3).

**Supplementary Table S3 | Taxon-specific primer sets and their annealing temperatures for quantitative PCR.**

| Target group | Primer | Sequence (5´- 3´) | Annealing temperature (°C) | Reference |
| --- | --- | --- | --- | --- |
| *Pseudomonas* sp. | Ps-for | GGTCTGAGAGGATGATCAGT | 63 | Tan et al. 2010 |
|  | Ps-rev | TTAGCTCCACCTCGCGGC |  | Tan et al. 2010 |
| *Fusarium oxysporum* | ITS1-F | CTTGGTCATTTAGAGGAAGTAA | 58 | Lievens et al. 2005 |
|  | AFP308R | CGAATTAACGCGAGTCCCAA |  | Lievens et al. 2005 |

**References**

1. Tan, Y. & Ji, G. Bacterial community structure and dominant bacteria in activated sludge from a 70 degrees C ultrasound-enhanced anaerobic reactor for treating carbazole–containing wastewater. *Bioresour. Technol*. **101**, 174–180 (2010).
2. Lievens, B., *et al*. Quantitative assessment of phytopathogenic fungi in various substrates using a DNA macroarray. *Environ*. *Microbiol*. **7**, 1698–1710 (2005).
